# Efficacy of species-specific protein antibiotics in a murine model of acute *Pseudomonas aeruginosa* lung infection

**DOI:** 10.1038/srep30201

**Published:** 2016-07-22

**Authors:** Laura C. McCaughey, Neil. D. Ritchie, Gillian R. Douce, Thomas J. Evans, Daniel Walker

**Affiliations:** 1 Institute of Infection, Immunity and Inflammation, College of Medical, Veterinary and Life Sciences, University of Glasgow, Glasgow, G12 8TA, UK; 2 The ithree institute, University of Technology Sydney, Ultimo, New South Wales, Australia; 3 Department of Biochemistry, University of Oxford, South Parks Road, Oxford, UK

## Abstract

Protein antibiotics, known as bacteriocins, are widely produced by bacteria for intraspecies competition. The potency and targeted action of bacteriocins suggests that they could be developed into clinically useful antibiotics against highly drug resistant Gram-negative pathogens for which there are few therapeutic options. Here we show that *Pseudomonas aeruginosa* specific bacteriocins, known as pyocins, show strong efficacy in a murine model of *P. aeruginosa* lung infection, with the concentration of pyocin S5 required to afford protection from a lethal infection at least 100-fold lower than the most commonly used inhaled antibiotic tobramycin. Additionally, pyocins are stable in the lung, poorly immunogenic at high concentrations and efficacy is maintained in the presence of pyocin specific antibodies after repeated pyocin administration. Bacteriocin encoding genes are frequently found in microbial genomes and could therefore offer a ready supply of highly targeted and potent antibiotics active against problematic Gram-negative pathogens.

For Gram-negative pathogens such as *Pseudomonas aeruginosa, Klebsiella pneumoniae* and *Escherichia coli* therapeutic options are often limited. In the case of the opportunistic pathogen *P. aeruginosa,* resistance to every available class of antibiotic has been observed and between 18% and 25% of clinical isolates are multidrug resistant^1^,^2^,^3^,^4^. In addition to inherent and acquired antibiotic resistance mechanisms, the ability of *P. aeruginosa* to form biofilms during chronic infection and the appearance of antibiotic resistant phenotypic variants during prolonged antibiotic therapy can render this pathogen essentially untreatable^5^,^6^,^7^. This is now evident with an increasing prevalence of pan-drug resistant *P. aeruginosa* infections worldwide^8^. Consequently, there is an urgent need to consider alternative strategies for antibiotic development, to bolster a developmental pipeline that in recent decades has yielded no effective novel small molecule antibiotics against *P. aeruginosa* and other difficult to treat Gram-negative bacteria^9^,^10^,^11^.

One alternative strategy for the discovery of effective antibiotics is the exploitation of potent narrow-spectrum antibiotics produced by many bacteria for intraspecies competition. In *P. aeruginosa, K. pneumoniae* and *E. coli* these take the form of multi-domain protein antibiotics known as the S-type pyocins, klebicins and colicins respectively^12^,^13^,^14^,^15^. These bacteriocins have evolved to efficiently cross the Gram-negative outer membrane through the parasitisation of active nutrient uptake pathways, which are an Achilles’ heel for Gram-negative bacteria^16^,^17^,^18^,^19^,^20^,^21^. The pyocins of *P. aeruginosa* target several different outer membrane receptors involved in uptake of the essential nutrient iron and pyocin L1 has also been shown to bind to the common polysaccharide antigen (CPA), which is the major surface antigen produced by *P. aeruginosa* when growing in the lungs of CF patients^14^,^22^,^23^,^24^. The cellular targets of bacteriocins are highly conserved, with cytotoxic activity most commonly taking the form of a nuclease activity targeting DNA, rRNA or tRNA, a pore-forming activity targeting the cytoplasmic membrane or an enzymatic activity targeting peptidoglycan synthesis^13^,^14^. Pyocins S1, S2, S3 and AP41 display DNase activity, pyocin S4 is a tRNase, pyocin S5 is a pore-forming toxin, pyocin S6 is an rRNase and pyocin M degrades lipid II^14^,^25^,^26^. For the recently described lectin-like pyocin L1 the cytotoxic mechanism is unknown. Their potency, targeting of essential nutrient uptake systems and active uptake across the outer membrane makes them ideal antibiotic candidates for the treatment of *P. aeruginosa* infection.

In this work we show that pyocin S2, pyocin AP41, pyocin S5 and pyocin L1 delivered directly to the murine lung display strong efficacy against diverse strains of *P. aeruginosa* in a murine model of acute *P. aeruginosa* lung infection. Furthermore, pyocin S5 is several orders of magnitude more potent than tobramycin and also offers protection against a lethal *P. aeruginosa* infection in the presence of pyocin S5-specific antibodies.

## Results

### Pyocins are stable in the murine lung

**Figure 1:**
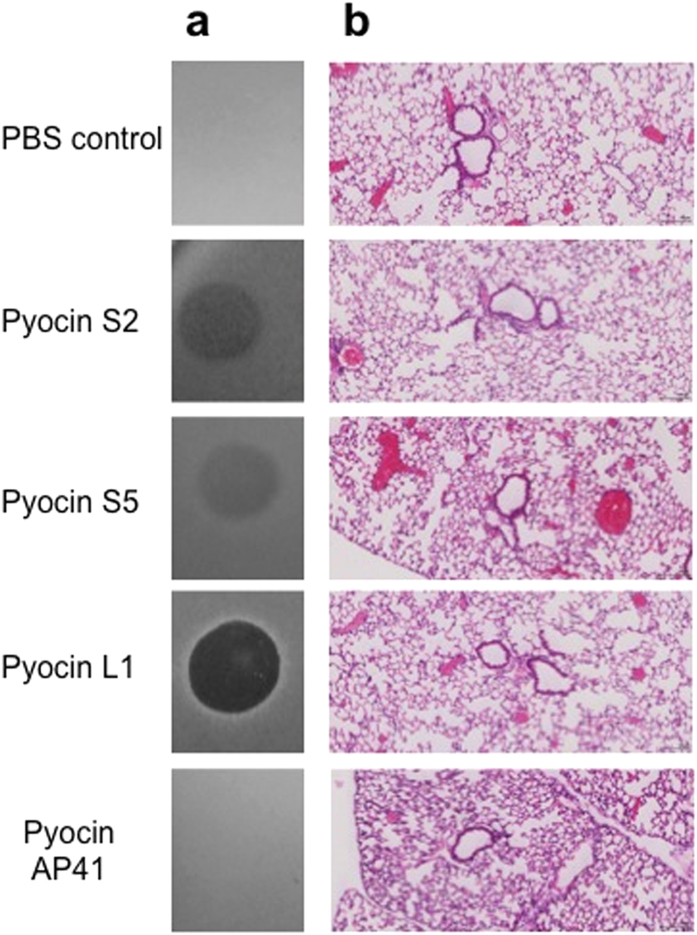
Pyocins are stable and do not cause inflammation or tissue damage in the murine lung. (**a**) Spot tests to determine stability of pyocins (75 μg) in lung tissue (24 h after administration) against *P. aeruginosa* P8 (*P. aeruginosa* P17 for pyocin S2). Five microlitres of homogenised post-caval lobe lung section in 100 μl of PBS was spotted onto a growing lawn of *P. aeruginosa*. The presence of clear zones indicates pyocin activity in the lung tissue and that pyocins are stable and still active *in vivo* for 24 h. (**b**) Hematoxylin and eosin (H&E) staining of paraffin-embedded sections of pyocin (75 μg) treated lung. Lack of inflammation or neutrophil influx are noted. All magnifications ×10.

To determine if pyocins can be effectively delivered to the lungs and are stable in this environment, recombinant pyocins S2, S5, AP41 and L1 (75 μg), were administered intranasally to healthy C57/BL6 mice (n = 3). After a 24 h incubation period, the postcaval lobe was removed from treated mice, homogenised and tested for the presence of active pyocin. Killing of *P. aeruginosa* was detected in lung homogenates from pyocin L1, S2 and S5 treated mice. Activity for pyocin AP41 was not detected, which could indicate this pyocin may be degraded *in vivo* (Fig. 1a). To investigate the effects of a single administration of pyocin on the host, pyocins S2, S5, AP41 and L1 (75 μg) were administered intranasally and after 24 h pyocin treated lungs were fixed using formalin (n = 4). Lung tissues visualised using hematoxylin and eosin staining were then scored for peribronchial infiltrate and alveolar macrophage involvement. The pyocin treated lungs showed no evidence of inflammatory infiltrate and were indistinguishable from the PBS treated lungs (Fig. 1b).

### Pyocins can afford protection against lethal *P. aeruginosa* infections

**Figure 2:**
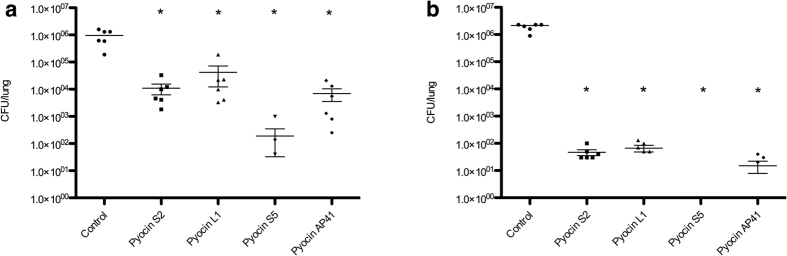
*P. aeruginosa* P8 bacterial recovery from pyocin treated mice. All mice were given 75 μg of pyocin. Bacterial counts were determined by CFU counts of homogenised lungs. (**a**) Mice treated with pyocin 1 h post-infection, all mice culled 4.5 h post-infection. (**b**) Mice treated with pyocin 1 h post-infection, pyocin treated mice survived to 24 h. No colonies were recovered from pyocin S5 treated mice in (**b**). Bars represent Mean ± SEM. *Denotes statistical significance for comparison of treatment versus control by a one-sided Mann-Whitney U test with Bonferroni correction applied.

To determine if pyocins are sufficiently active in the lung to reduce bacterial load, pyocins S2, S5, AP41 and L1 (75 μg) or PBS for control mice, were administered intranasally 1 h post-infection with a normally lethal dose (approx 1 × 10^7^ CFU) of *P. aeruginosa* P8, a non-mucoid aztreonam-resistant isolate from a cystic fibrosis (CF) patient. In these experiments mice were culled at 4.5 h post-infection and bacterial counts from lung homogenates were compared to PBS-treated controls. Pyocin S5 showed greatest efficacy in reducing bacterial numbers and viable bacteria were recovered from only three out of six pyocin S5 treated mice (Fig. 2a). Pyocins L1, S2, and AP41 significantly reduced the bacterial load by approximately 20-, 80- and 130-fold, respectively (Fig. 2a). This experiment was repeated and again all pyocin treated groups showed significantly reduced bacterial counts (Figure S1a).

To determine if pyocin treatment affords protection against a lethal *P. aeruginosa* infection, mice (n = 6) were similarly infected with *P. aeruginosa* P8 and treated 1 h post-infection with pyocins S2, S5, AP41 and pyocin L1 (75 μg). All PBS-treated mice were culled at 4.5 h post-infection and all pyocin treated mice survived to the endpoint of the experiment at 24 h. In this experiment, pyocin S5 again showed the greatest efficacy with no bacteria recovered from any of the six pyocin S5 treated mice. In addition, pyocins S2, L1 and AP41 were also highly effective in this model significantly reducing bacterial counts in excess of 4-log units (Fig. 2b). This experiment was repeated, all pyocin treated mice survived to 24 h and bacterial counts were similarly significantly reduced (Figure S1b). Thus, pyocins are highly effective in reducing bacterial load in the lung and are able to afford protection against a lethal *P. aeruginosa* infection when administered post-infection. In similar experiments in which mice were pre-treated with pyocin 6 h prior to infection, pyocins were shown to be similarly effective in reducing bacterial numbers and in protecting against a normally lethal *P. aeruginosa* infection (Figure S2a,b).

Since strains of *P. aeruginosa* are phenotypically diverse, we tested the efficacy of the pyocins against three additional isolates. Including *P. aeruginosa* P8 the phenotypes covered, using the minimal number of strains so as to reduce animal numbers, were mucoid, non-mucoid, drug-resistant, environmental and clinical: *P. aeruginosa* P17 and *P. aeruginosa* P5 (mucoid), both from CF patients and *P. aeruginosa* E2, an environmental isolate. Pyocin S2 was not active against *P. aeruginosa* P5 or *P. aeruginosa* E2 *in vitro*, therefore was not used to treat these strains *in vivo* and similarly pyocin L1 was not used against *P. aeruginosa* P17. Pyocin S5, L1 and S2 treated mice infected with *P. aeruginosa* P17, P5 or E2 all survived until the endpoint of the experiment (24 h) and viable bacteria counts were either reduced to low levels or absent (Table S1). In contrast, treatment of *P. aeruginosa* E2 with pyocin AP41 failed to afford protection and these mice were culled at 5.5 h post-infection. However, pyocin AP41 treatment was successful for *P. aeruginosa* P5 infected mice and for five out of six of the *P. aeruginosa* P17 infected mice. Thus, pyocins show strong efficacy against diverse strains of *P. aeruginosa* with pyocin S5 treatment displaying the largest effect on reducing bacterial load.

### Pyocin tolerance and mitigation strategies

**Figure 3:**
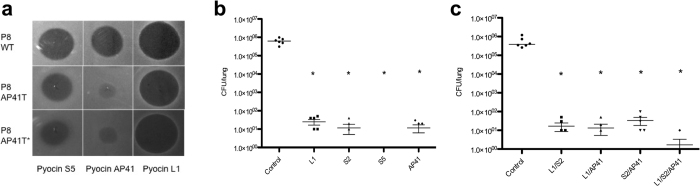
Acquired tolerance to pyocins can be overcome by treating with a range of pyocins. (**a**) Activity of pyocins S5, AP41 and L1 against WT P8, P8AP41T (a pyocin AP41 tolerant P8 strain) and P8AP41T* (P8AP41T recovered from untreated control mice shown in b). Purified protein at 200 μg ml^−1^ was spotted onto a growing lawn of bacteria. Clear zones indicate pyocin cytotoxicity. (**b**) Bacterial counts for mice infected with P8AP41T and treated 1 h post-infection with pyocins (75 μg). Pyocin treated mice survived to 24 h. No colonies were recovered from pyocin S5 treated mice. Bars represent Mean ± SEM. *Denotes statistical significance for comparison of treatment versus control by a one-sided Mann-Whitney U test with Bonferroni correction applied. (**c**) P8 infected mice treated 1 h post-infection with pyocin combinations (7.5 μg of each pyocin); pyocin treated mice survived to 24 h. Bacterial counts were determined by CFU counts from homogenised lungs. Bars represent Mean ± SEM. *Denotes statistical significance for comparison of treatment versus control by a one-sided Mann-Whitney U test with Bonferroni correction applied.

To determine if pyocin tolerance or resistance was acquired upon pyocin treatment *in vivo,* viable bacteria recovered from mice that survived infection to the 24 h end-point, in all acute infection experiments discussed in this work, were tested for pyocin susceptibility. From these experiments no pyocin resistant colonies were isolated. However, we obtained a single isolate (P8AP41T) from a pyocin AP41 treated mouse that showed increased tolerance (approximately 1000-fold) to pyocin AP41. The sensitivity of P8AP41T to pyocins S5 and L1 was similar to the parent strain P8 (Fig. 3a). *In vivo* mice (n = 6) infected with P8AP41T and treated with pyocins L1, S2, S5 and AP41 survived until the endpoint of the experiment at 24 h and bacterial numbers were significantly reduced from lung homogenates relative to PBS-treated controls, which were culled at 6 h post-infection (Fig. 3b). Thus, a pyocin AP41-tolerant mutant can still be successfully treated with pyocin AP41 at high concentrations despite pyocin susceptibility testing showing that this strain remained tolerant to pyocin AP41 during infection (Fig. 3a).

As all four pyocins used in this study parasitise different receptors in *P. aeruginosa* an obvious strategy to prevent the occurrence of pyocin resistance is to use ‘pyocin cocktails’ consisting of two or more pyocins in combination. The following pyocin combinations were tested: L1/S2, L1/AP41, S2/AP41 and L1/S2/AP41 with 7.5 μg of each pyocin. PBS control mice were culled 4.5 h post-infection and all pyocin treated mice survived until 24 h. Viable bacteria were recovered at a low level from pyocin treated mice and for the combination of L1/S2/AP41, bacteria were recovered from only one of six treated mice, indicating that pyocin combinations show enhanced efficacy over the use of individual pyocins (Fig. 3c).

### Pyocin S5 shows improved killing of *P. aeruginosa* in the murine lung compared to tobramycin

**Figure 4:**
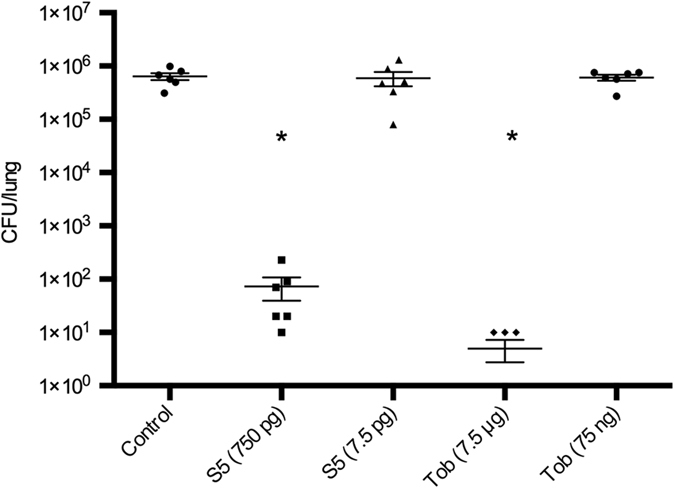
Comparison of pyocin S5 and tobramycin efficacy. Bacterial counts were determined by CFU counts of homogenised lungs. (**a**) Mice treated 1 h post-infection, S5–750 pg and tobramycin–7.5 μg mice survived to 24 h. All other mice culled 5.5 h post-infection. Bars represent Mean ± SEM. *Denotes statistical significance for comparison of treatment versus control by a one-sided Mann-Whitney U test with Bonferroni correction applied.

To compare pyocin efficacy directly with a current frontline treatment, we compared pyocin S5 with tobramycin, which is the most commonly used inhaled treatment for *P. aeruginosa* lung infection in patients with CF. To determine the relative potency of pyocin S5 compared to tobramycin, *P. aeruginosa* P8 infected mice (n = 6) were treated with pyocin S5 (750 pg or 7.5 pg) or tobramycin (7.5 μg or 75 ng). Groups treated with 750 pg of pyocin and 7.5 μg of tobramycin survived to 24 h and had significantly reduced bacterial counts compared to the PBS controls (Fig. 4). All other groups were culled 5.5 h post-infection due to the severity of the infection. These results show that the lowest dose tested at which pyocin S5 is effective was 750 pg and the lowest dose tested at which tobramycin is effective was 7.5 μg. Therefore, pyocin S5 is between 100-fold and 1,000,000-fold more potent than tobramycin in this model of infection.

After ascertaining that pyocin S5 is effective in this model at a concentration lower than 1 nM, we tested the efficacy of pyocins S2, L1 and AP41 at lower concentrations than previously used (7.5 μg and 750 ng). Against *P. aeruginosa* P8, the minimum effective dose of pyocins S2 and AP41 is ≤750 ng and the minimum effective dose of pyocin L1 is between 7.5 μg and 750 ng (Table S2).

### Pyocin S5 can afford protection against lethal *P. aeruginosa* infections in the presence of pyocin S5 antibodies

To ascertain if repeated exposure to pyocins gives rise to an antibody response that is detrimental to treatment, mice were repeatedly exposed to high doses (75 μg) of pyocin S5 (100,000 times the effective therapeutic dose of pyocin S5 in this model). Pyocin S5 was administered three times, with two weeks between each administration, either via the intranasal route (I.N.) or the intraperitoneal (I.P.) route. Thirteen weeks after the first treatment, mice (n = 5) were infected intranasally with *P. aeruginosa* P8 (I.N. group infected with 1.4 × 10^7^ CFU; I.P. group infected with 5.0 × 10^6^ CFU) and treated intranasally 1 h post-infection with 75 μg of pyocin S5 or PBS. A control group administered only PBS, intranasally prior to infection was also included.

**Figure 5:**
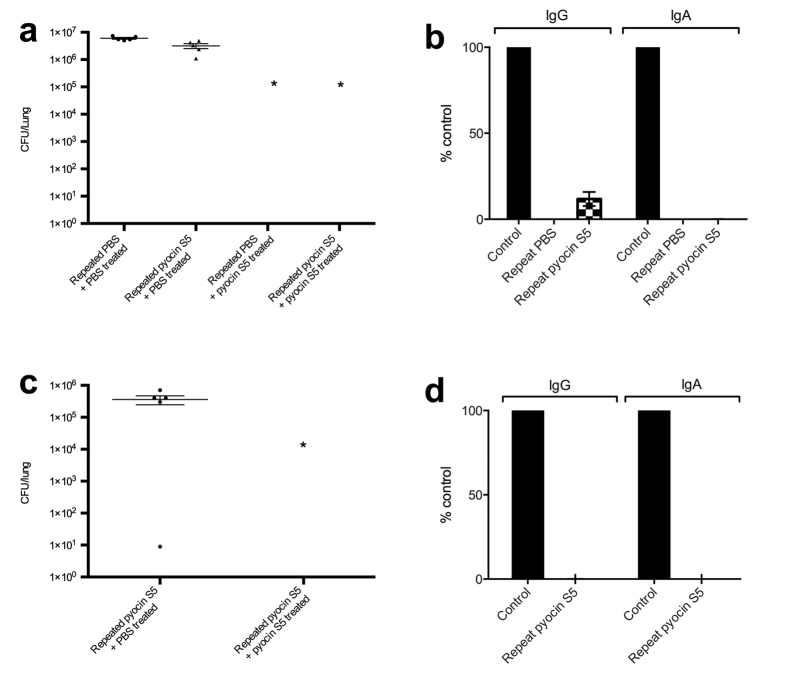
Pyocin S5 can afford protection against lethal *P. aeruginosa* infections in the presence of pyocin S5 antibodies. (**a**) Bacterial counts were determined by CFU counts from homogenised lungs. Multiple doses of pyocin S5 (75 μg/dose) were administered intranasally three times, two weeks apart over four weeks. At thirteen weeks, mice were infected with *P. aeruginosa* P8 and treated with pyocin S5 (75 μg) or PBS intranasally 1 h post-infection. Bars represent Mean ± SEM of counts from 5 animals. *Denotes statistical significance for comparison of treatment versus control by a one-sided Mann-Whitney U test with Bonferroni correction applied. (**b**) Pyocin S5-specific IgG and IgA serum levels for mice repeatedly exposed to pyocin S5 or PBS (as described in a). The control group were immunized subcutaneously (S.C.) with pyocin S5 (75 ug/dose) in Freunds complete/incomplete adjuvant on three occasions, two weeks apart. No pyocin S5-specific IgA was detected in any of the animals tested. Bars represent Mean ± SEM calculated from the serum of 5 animals per group. (**c**,**d**) as for (**a**,**b**) except mice were repeatedly exposed to pyocin S5 via the intraperitoneal (I.P.) route prior to intranasal pyocin S5 or PBS treatment. The pyocin S5-specific IgG levels in (**d**) were very low in the pyocin S5 only group (1000-fold less than the Freunds complete/incomplete control group) and no pyocin S5-specific IgA was detected.

For the I.N. groups, all pyocin S5 treated mice survived to the 24 h time-point, while all PBS-treated mice were culled 5 h post-infection due to severity of symptoms. The bacterial load of the lungs was determined and no viable bacteria were recovered from any of the pyocin S5 treated mice (Fig. 5a). The levels of pyocin-S5 specific IgG and IgA were analysed for each mouse. There were no pyocin S5-specific IgA antibodies detected in these mice however, there were low levels of pyocin S5-specific IgG present in the mice previously exposed to pyocin S5 (10-fold less than that observed in control animals which were immunized with pyocin S5 using Freunds complete/incomplete adjuvant) (Fig. 5b).

All mice exposed to pyocin S5 via the I.P. route and whose infection was subsequently treated with pyocin S5 survived to the 24 h time-point. In contrast, animals exposed to pyocin S5 parenterally whose infection was treated with PBS were culled 5 h post-infection due to the severity of symptoms. As described above, no viable bacteria were recovered from the lungs of pyocin S5 treated mice (Fig. 5c). The pyocin S5-specific IgG levels were very low in the pyocin S5 only group (1000-fold less than the Freunds complete/incomplete control group) and no pyocin S5-specific IgA was detected (Fig. 5d). Thus, repeated administration of pyocin S5 at 100,000 times its effective dose either by the I.N. or I.P. routes was very poorly immunogenic, with only small amounts of pyocin-specific IgG being produced. Moreover, even in the presence of these low levels of antibody, pyocin S5 remained highly effective in treating bacterial infection.

## Discussion

The development of bacteriocins as novel therapeutics for the treatment of problematic Gram-negative bacteria has been widely discussed over the last decade^14^,^27^,^28^,^29^,^30^,^31^. In this work, several different *P. aeruginosa*–specific pyocins were highly effective at reducing bacterial load and affording protection in a lethal *P. aeruginosa* model of acute pneumonia when delivered directly to the murine lung. Pyocins remained stable and retained activity for a minimum of 24 h in this location. Moreover, pyocin-specific antibody production did not appear to affect the efficacy of the aforementioned pyocin.

In addition to their potency, the species-specificity of these antibiotics is a desirable trait for two reasons. Firstly, unlike broad-spectrum antibiotics, the lack of selective pressure will limit the development of antibiotic resistance within the microbial population and secondly, the narrow spectrum of killing provides opportunities to successfully treat bacterial infections whilst leaving the normal bacterial flora intact. Well-established complications associated with antibiotic induced dysbiosis include antibiotic-associated diarrhea and *Clostridium difficile* infection^32^,^33^. More recently, microbial imbalances have been suggested to play a role in a range of chronic diseases such as Crohn’s disease, diabetes, obesity and rheumatoid arthritis^34^,^35^,^36^,^37^. The ability to maintain a healthy gut while undergoing antibiotic treatment may not only improve the health of patients, but also the outcome of treatment.

Of the pyocins evaluated in this study, the receptors for pyocins S2 and S5 are known to be the TonB-dependent iron-siderophore receptors FpvAI and FptA^16^,^17^, respectively, whilst pyocin L1 has been shown to bind the common polysaccharide antigen (CPA) of *P. aeruginosa* lipopolysaccharide^22^. The receptor for pyocin AP41 remains to be identified. FptA and the CPA are known to be widely distributed among strains of *P. aeruginosa*^23^,^24^,^38^,^39^ and the detection of pyoverdine in the sputa of CF patients suggests that pyoverdine and its receptors FpvAI and FpvAII, play a key role in the infection process in humans^40^,^41^.

Over the last decade pharmaceutical companies have focused on a post-genomic, target driven antibiotic discovery program, with little success and very few small molecule antibiotics making it to market. Most small molecule antibiotics currently used in clinical practice came from the identification and modification of naturally occurring antibiotics. Therefore, returning to the exploitation of natural products is likely to be a more successful route to developing novel antimicrobials.

## Methods

### Cloning and purification of pyocins

The genes encoding pyocin AP41 and its immunity protein (ImAP41) were amplified from the genomic DNA of *P. aeruginosa* C763 by PCR using primers designed to introduce an NdeI site at the start of the pyocin encoding gene and an XhoI in place of the stop codon of the ImAP41 encoding gene. The PCR product was digested with NdeI and XhoI and ligated into the corresponding sites of the *E. coli* expression vector pET21a to give pETPyoAP41, which encodes pyocin AP41-ImAP41 with a C-terminal His_6_-tag on the immunity protein. The gene encoding pyocin S5 was similarly amplified from the genomic DNA of strain PAO1 and the digested PCR product ligated into pET15b to give pETPyoS5, which encodes pyocin S5 with an N-terminal His_6_-tag. Pyocins AP41 and S5 were overexpressed from *E. coli* BL21(DE3)pLysS carrying the relevant plasmid with initial purification by nickel affinity chromatography. Remaining contaminants were removed by gel filtration chromatography on a Superdex S200 26/60 column (GE Healthcare). Pyocin L1 and the pyocin S2-ImS2 complex were purified as described previously^22^,^27^. Contaminating lipopolysaccharide was removed using 1 ml gravity flow endotoxin removal columns (Thermo Scientific) and proteins were filter sterilised using a 0.2 μM syringe filter.

### Pyocin sensitivity assays

One hundred and fifty microlitres of test strain culture at OD_600_ = 0.6 was added to 6 ml of 0.8% agar and poured over an LB agar plate. Five microlitres of each bacteriocin or lung homogenate at varying concentrations was spotted onto the plates and incubated for 24 h at 37 °C.

### Ethics statement

All animal experiments were performed in accordance with the UK Animals (Scientific procedures) Act, authorized under a UK Home Office License and all procedures were approved by the animal project review committee of the University of Glasgow. The project license number assigned by the animal project review committee of the University of Glasgow was 60/4361.

### *In vivo* pyocin efficacy

For all experiments, six week-old, female, pathogen-free C57/BL6 mice weighing 15–21 g were used (Charles Rivers Laboratories, UK). All animals received food and water *ad libitum* and experimental group sizes were calculated from preliminary experiments (data not shown), which indicated that statistical significance could be determined using groups of 3 to 6 animals. Experimental endpoints were determined by a clinical scoring system (based on a number of physical and behavioral attributes) or culled at the pre-determined 24 h time point. All mice were culled by carbon dioxide asphyxiation. For intranasal delivery, animals were lightly anesthetized using isofluorothane. Mice were inoculated intranasally with 25 μl of bacterial culture containing approximately 1 × 10^7^ CFU of the selected *P. aeruginosa* strain and/or 25 μl of pyocin/tobramycin by direct application of the solution to both nostrils (12.5 μl per nare). The dose of pyocin/tobramycin used was based on mg/kg of the antibiotics in a 25 g mouse.

### Histology

Lungs were fixed *in situ* using 10% formalin solution before being removed and placed in fixative. Histology processing and hematoxylin and eosin (H&E) staining was carried out by the Veterinary Diagnostic Services Laboratory at the University of Glasgow. High-resolution whole slide images were captured on the Leica SCN400 slide scanner and slides were scored blind by two independent assessors for peribronchial infiltrate and alveolar involvement.

### Repeated pyocin exposure

Pyocin S5 or PBS was given three times, two weeks apart with administration either via intranasal route (referred to as I.N. groups) or intraperitoneal route (referred to as I.P. groups). For I.N. administration the groups were: PBS and pyocin S5 (75 μg; 25 μl at 3 mg ml^−1^). For I.P. administration the group was pyocin S5 (75 μg; 100 μl at 750 μg ml^−1^). Thirteen weeks after the first exposure mice (n = 5) were infected with *P. aeruginosa* P8 (I.N group infected with 1.4 × 10^7^ CFU, I.P group infected with 5.0 × 10^6^ CFU) and treated intranasally one hour post-infection with 75 μg of pyocin S5 or PBS, as described previously. For analysis of IgG and IgA responses, blood was obtained via cardiac puncture immediately after carbon dioxide asphyxiation. Serum was stored at −80 °C. For ELISAs Greiner 96-well plates (MaxiSorp) were coated with purified recombinant pyocin S5 (7.5 μg ml^−1^, 50 μl/well) protein in PBS overnight at 4 °C. The plates were washed three times with phosphate buffered saline +0.05% TWEEN20 (PBST) and then blocked for 1 h at 37 °C with 150 μl of blocking buffer (1% bovine serum albumin (BSA) in PBS). After washing, five-fold serially diluted samples were added, starting at a dilution of 1/50 in blocking buffer, and incubated for 2 h at 37 °C. Serum from mice given pyocin S5 + Freunds complete/incomplete subcutaneously three times over four weeks was used as a positive control on each plate. After washing with PBST, 50 μl of anti-mouse IgG (Fc specific)–peroxidase antibody ((1/1000 dilution) Sigma, UK) or anti-mouse IgA (α-chain specific)−peroxidase antibody ((1/250 dilution) Sigma, UK) in PBST/0.1%, BSA was added and plates were incubated for 1 h at 37 °C. Plates were developed using SIGMA*FAST* OPD (*o*-Phenylenediamine dihydrochloride) tablets (Sigma, UK).

### Statistics

Due to small sample sizes non-parametric tests were used for analysis. The Kruskal-Wallis one-way analysis of variance method was used to test if samples originated from the same distribution. One-sided Mann-Whitney U tests with a significance threshold of P ≤ 0.05, adjusted for multiple comparisons using the Bonferroni correction, was then used to analyse the specific sample pairs for significant differences. All mice, including outliers were included in the statistical analysis.

## Additional Information

**How to cite this article**: McCaughey, L. C. *et al*. Efficacy of species-specific protein antibiotics in a murine model of acute *Pseudomonas aeruginosa* lung infection. *Sci. Rep.* **6**, 30201; doi: 10.1038/srep30201 (2016).

## Supplementary Material

Supplementary Information
