# Supplementary material for: Efficacy of species-specific protein antibiotics in a murine model of acute Pseudomonas aeruginosa lung infection
Source: Sci Rep. 2016 Jul 22;6:30201. doi: 10.1038/srep30201 (PMC4957109; doi:10.1038/srep30201)
Supplement: Supplementary Information [file srep30201-s1.pdf]

Efficacy of species-specific protein antibiotics in a murine model of acute *Pseudomonas aeruginosa* lung infection

Laura C. McCaughey, Neil. D. Ritchie, Gillian R. Douce, Thomas J. Evans, Daniel Walker

Supplementary information

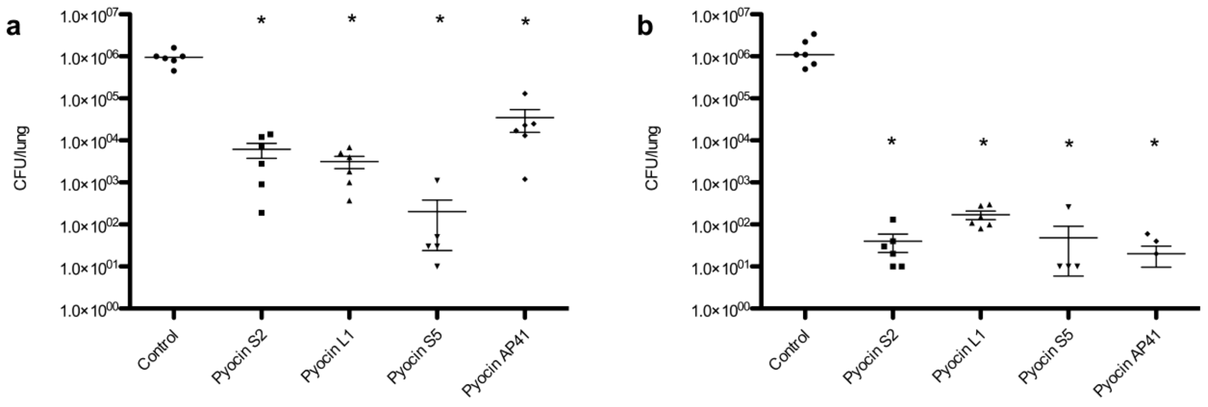

Figure S1. **Biological repeats of experiments in Figures 2 (c) and (d).** *P. aeruginosa* P8 bacterial recovery from pyocin treated mice. All mice were given 75 µg of pyocin. Bacterial counts were determined by CFU counts of homogenised lungs. (a) Mice treated with pyocin 1 h post-infection, all mice culled 4.5 h post-infection. (b) Mice treated with pyocin 1 h post-infection, pyocin treated mice survived to 24 h. Bars represent Mean ± SEM. * denotes statistical significance for comparison of treatment versus control by a one-sided Mann-Whitney U test with Bonferroni correction applied.

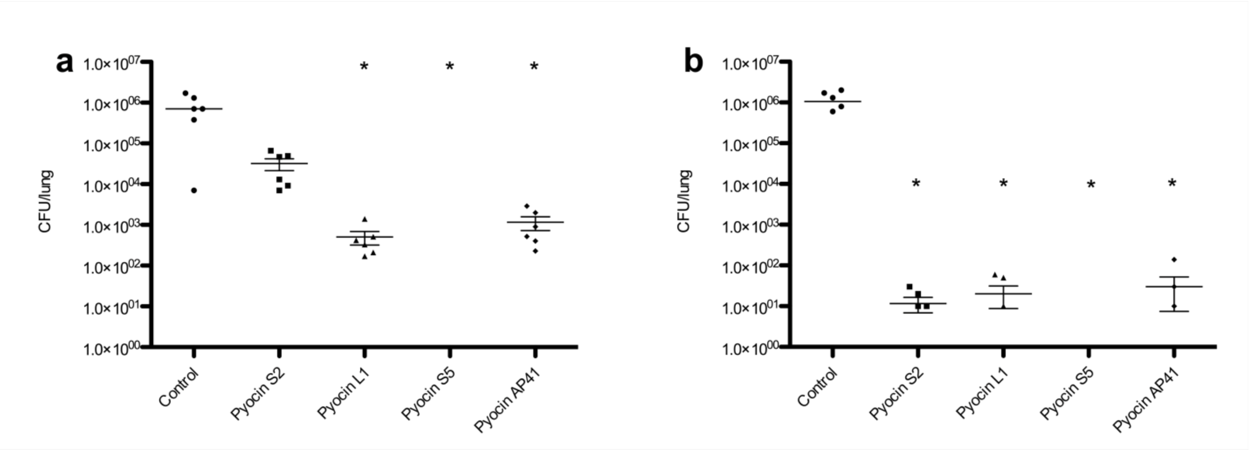

Figure S2. ***P. aeruginosa*** **P8 bacterial recovery from pyocin treated mice.** All mice were given 75 µg of pyocin. Bacterial counts were determined by CFU counts of homogenised lungs. (a) Mice treated with pyocin 6 h pre-infection, all mice culled 4 h post-infection. (b) Mice treated with pyocin 6 h pre-infection, pyocin treated mice survived to 24 h. No colonies were recovered from pyocin S5 treated mice in a) and b). Bars represent Mean ± SEM. ∗ denotes statistical significance for comparison of treatment versus control by a one-sided Mann-Whitney U test with Bonferroni correction applied.

	Infecting *P. aeruginosa* strain		
**Treatment**	**P5**	**P17**	**E2**
No treatment	$1.7x10^5$ CFU/lung	$4.4x10^5$ CFU/lung	$1.5x10^5$ CFU/lung
Pyocin L1	40	X	No colonies detected
Pyocin S2	X	No colonies detected	X
Pyocin AP41	No colonies detected	No colonies detected ✚	$1.3x10^4$ CFU/lung ★
Pyocin S5	No colonies detected	No colonies detected	No colonies detected

Table S1. Bacterial counts for pyocin treated *P. aeruginosa* isolates. Mice were infected with a lethal dose of *P. aeruginosa*. Untreated mice were culled 4 h - 5.5 h post infection. Pyocin treated mice (75 µg) survived to 24 h. ★Mice culled at same time as control.✚1 mouse coughed up pyocin AP41 treatment and was culled at 4 h post-infection (bacterial count $1.3x10^5$ CFU/lung). X - Pyocin was not used against this strain.

**Treatment**	**Lowest active dose tested**	**Corresponding molarity**
Pyocin L1	750 ng	1.06 µM
Pyocin S2	750 ng	358 nM
Pyocin AP41	750 ng	319 nM
Pyocin S5	750 pg	535 pM
Tobramycin	7.5 µg	641 µM

Table S2. Minimum dose of pyocin tested that affords protection against *P. aeruginosa* P8 infection. The lowest active dose tested represents the lowest dose tested with which the treated mice survived to 24 h.
